# The Influence of Recreational Hiking on the Prevalence of Cardiovascular and Psychiatric Diseases Among Population of Republic of Serbia

**DOI:** 10.3390/healthcare13060680

**Published:** 2025-03-20

**Authors:** Milos Gostimirovic, Jovana Rajkovic, Ana Bukarica, Ljiljana Gojkovic-Bukarica

**Affiliations:** 1 Institute for Pharmacology, Clinical Pharmacology and Toxicology, Faculty of Medicine, University of Belgrade, 11000 Belgrade, Serbia; jolarajkovic@yahoo.com (J.R.); ana.bukarica@institutdedinje.org (A.B.); bukarica@rcub.rs (L.G.-B.); 2 Institute for Cardiovascular Diseasses Dedinje, Faculty of Medicine, University of Belgrade, 11000 Belgrade, Serbia

**Keywords:** recreational hiking, mental health, cardiovascular diseases, pharmacotherapy, chronic disease prevention

## Abstract

**Background:** Hiking is a physical activity recommended for people of all ages. In an era of increased incidence of cardiovascular and psychiatric diseases, directing individuals to hiking can be very important from both public health and socioeconomic perspectives. Since the health status of recreational hikers and the general population in the Republic of Serbia has not been compared yet, our objectives are to compare the health-related characteristics of those two groups, including the prevalence of comorbidities, pharmacotherapy, and drug consumption. **Methods:** A descriptive epidemiological study was conducted. Research questions were asked via two specially prepared questionnaires distributed through the Google Forms platform. The means of the two groups were tested by a two-sample Student *t*-test for independent variables. **Results:** The sample consisted of 259 hikers and 292 people from the general population. A total of 199 hikers (76.8%) and 218 people from the general population group (74.7%) were declared as healthy. The statistically significant differences between the groups included age, sex, education level, and body mass index. In both groups, the majority of those with pre-existing medical conditions had at least one cardiovascular disease (23.5% of the hikers and 19.5% of the individuals in the general group). Pre-existing psychiatric diseases were noted in 6% of the hikers and in 12% of those in the general group. The average durations of the disease in the hiker and general population were 11.9 and 8.4 years, respectively (*p* < 0.05), whereas, there were no differences in drug consumption. **Conclusions:** This pilot study represents the comparison of the cardiovascular and mental health among hikers and the general population in the Republic of Serbia. Although psychiatric diseases were clearly less prevalent among hikers, the prevalence and burden of cardiovascular diseases must be interpreted with caution, due to big age difference between the respondents from both groups. However, our future studies will employ objective measurements and clinical parameters rather than self-reported surveys, so that the health benefits of hiking appear more clearly.

## 1. Introduction

Regular physical activity (PA) is known to reduce the risk of many diseases, including cardiovascular, metabolic, and psychosomatic diseases [1,2]. Proposed mechanisms include its action on the heart (improvement in cardiac mitochondrial biogenesis), blood vessels (decrease in the plaque formation/increased eNOS expression), and blood (increased oxygen carrying capacity) [3]. Moreover, participation in sports seems to promote benefits to cardiovascular structure and function in adolescents, mostly by reducing blood pressure and intima-media thickness of blood vessels [4]. In terms of mental health, exercise training improves neurobehavioral processes (working memory, cognitive control, conflict resolution, vigilance, and preservation) and a mood (through interoceptive awareness and inhibition of avoidance). This is probably due to improvement in synaptic plasticity, neurotransmitter, and cerebrovascular glial cell function [5].

Unfortunately, some external factors, such as the recent COVID-19 pandemic, may be linked with significant decreases in mobility, walking, and PA and increases in sedentary activity, which could negatively affect health. In a post-COVID-19 era, engaging in PA continues to be strongly encouraged [6]. When choosing the type and the intensity of PA, factors that must be taken into consideration include the person’s aspirations and capacities, additional beneficial effects (e.g., for mental health), and for example, the exercising environment.

In this context, hiking stands out as an excellent type of PA for several reasons: it is suitable for people of all ages, reduces anxiety, improves social interactions, reduces body mass index (BMI), preserves the vitality of blood vessels, brings people closer to nature, and raises the awareness of important environmental issues [7]. The positive effects of hiking on cardio-metabolic status include a reduction in systolic blood pressure, a reduction in the need for insulin in people with type 1 diabetes mellitus, the stabilization of serum cholesterol levels, improvement in bone density, and the maintenance of serum calcium levels [1]. In terms of psychiatric diseases (PDs), hiking releases adrenaline and endogenous endorphins that maintain mood and relieve anxiety [1,7]. With the exception of the risk of acute mountain sickness and dehydration due to heat stress, hiking is considered a generally safe activity [8,9]. In people with pre-existing metabolic syndrome, hiking at moderate or low altitude is considered both beneficial (improving cardiovascular parameters) and safe [10,11]. Moreover, some studies even suggest that people more engaged in regular mountaineering activities have lower risk for acute fatal cardiovascular events (e.g., sudden cardiac disease) during hiking activity [12].

Owing to all of the above, many international associations are engaged in the promotion of hiking through pamphlets encouraging the education of the population (e.g., the AHS (American Hiking Society)) [13].

Considering their impact on overall mortality and morbidity, cardiovascular diseases (CVDs) still represent a global public health problem [14]. According to data from the Institute for Public Health of the Republic of Serbia (RS), in 2021, 56,610 people died from some type of CVD, accounting for 40% of the total deaths that year. Moreover, an increase in CVD mortality in the RS has been evident during the past decade (763.6 and 828.3 per 100,000 people for the years 2012 and 2021, respectively), and the majority of these individuals were elderly [15]. Given the unfavorable epidemiology of CVDs in the RS, an important measure for reducing the burden of CVDs represents a health system that should work unhindered at all levels of healthcare. Unfortunately, the complications of advanced disease are more often addressed, so patients need hospital admissions with an uncertain outcome. This is why primary healthcare doctors have to recognize the risk factors for CVDs in their patients, which then must be influenced by timely advice and/or intervention [16,17,18]. In addition to excessive caloric intake and tobacco/alcohol consumption, modifiable factors include physical inactivity, which is increasingly prevalent in the modern age [19]. However, little is known about the impact of hiking on medication use and the reduction in the risk of CVDs and PDs, especially among the population in the RS.

The main hypotheses of this research are as follows:

- Recreational hikers have fewer risk factors for developing CVDs and PDs than the general population.
- Recreational hikers less frequently report history of pre-existing CVDs and/or PDs than the general population.
- Recreational hikers use fewer drugs (for a shorter time and with fewer side effects) and have a greater quality of life than the general population.

Therefore, the main goals of this work are to compare the following:

- The presence of socio-demographic and other risk factors for developing CVDs and PDs.
- The prevalence of different clinical entities in people with (pre-existing) CVDs and PDs.
- The frequency, duration, and side effects of chronic CVD and PD pharmacotherapy.
- The quality of life among the general population and recreational hikers in the RS.

## 2. Materials and Methods

An observational study was conducted. Research questions written in the Serbian language were asked via two specially prepared questionnaires. In the electronic form, questionnaires were distributed via the Google Forms platform and were available for the respondents from 19 January to 3 February 2023. The first questionnaire was intended for the population of hikers (H), whereas, the second questionnaire was intended for the general (G) population. The H group consisted of recreational hikers that were official members of various hiking clubs of the Mountaineering Association of Serbia (MAS). The link for this survey was placed in different sources: the official website of MAS, hikers’ e-mail addresses, different social media, and informal groups/chats on mobile phones. We also recruited participants from the in-person/live hiking activity that we participated in as well. For special occasions, we had this survey prepared in paper form. The department of marketing of the MAS also helped with the recruitment. The G group consisted of people who did not participate in hiking activities (were not members of the MAS). The recruitment process mostly took place at the Faculty of Medicine University of Belgrade, among students but also among members of their families (brothers, sisters, parents, grandparents, and other relatives). The initial recruitment of students was suitable due to their availability, motivation to participate as future medical workers, and presumably high level of self-consciousness about health-related topics. The students from other faculties and their families took place as well. Here, the survey link was placed mostly on social media and in the online groups of various student organizations.

The questions from both questionnaires related to demographic characteristics (gender, age, anthropometric measurements, education level, etc.), lifestyle and risk factors (BMI, PA, diet, consumption of central nervous system (CNS) active substances (alcohol), smoking, etc.), attitudes toward health (blood analysis controls, blood pressure measurements, regular visits to the doctor), the presence/absence of hypertension (HTN) and other diseases, and the use of medications (type of therapy, dosage regimen, co-medication, factors that lead to altered pharmacokinetics of the drug, and the potential effects of interactions between drugs). Additionally, questions from the first questionnaire were related to hiking experience (years of hiking, main reason/motivation for hiking, and health symptoms during the hiking action). The questions about PA were adapted from the internationally recognized International Physical Activity Questionnaire (October 2002). Questions regarding the frequency of PA with different intensity were adapted from the WHO definitions: light (burns less than 3.0 metabolic equivalent of task (METs), for example walking at a slower or leisure pace, cooking activities, or light household chores), moderate (burns 3.0 to 5.9 METs, for example brisk walking, dancing, water aerobics, hiking, or rollerblading), and intensive (vigorous) (burns more than 6.0 METs, for example swimming, running, biking, jumping rope, or gymnastics) [20].

The question on the self-assessment of the level of depressive mood was taken from the Kessler Psychological Distress Scale (K10) [21], Harvard Medical School, Boston, USA, and is a scale used to self-assess depressive and anxiety symptoms in the past 30 days. Other questions were adapted to specific objectives and were created by searching the available literature. Criteria for the inclusion in the research group (for both H and G) are as follows: respondent’s report of HTN and diabetes, according to the valid classification of the European Association for Hypertension (prehypertension, HTN grade I, II, III, isolated systolic HTN) and ADA (American Diabetes Association), and citizenship of the RS. An additional criterion for inclusion in the research (for the H group) was at least one year of hiking experience, membership in the MAS or related organization, age older than 18 years, and recreational PA, as mentioned above in the recruitment process section. The exclusion criteria for both groups were people with permanent residence outside the RS, underage, and professional athletes.

The research was approved on 12 January 2023, by the official Ethics Committee (consent numbers 17/I-26 and 17/I-27) (available upon request). During the aforementioned period, 259 hikers and 292 people from the general population completed the questionnaires, thus, they were considered participants in the research. The study conformed to the Good Clinical Practice guidelines, and the following procedures were used in accordance with the Helsinki Declaration. Informed consent was obtained from all individual participants included in the study. All the respondents were informed that their data were completely confidential.

The results are expressed via descriptive statistics, i.e., measures of a central tendency (arithmetic mean, $\bar{x}$ ± standard deviation (SD)). The data were imported to the SPSS Software v20. After confirming the normal distribution, means of the two groups were tested by a two-sample Student *t*-test for independent variables. In cases of >2 groups, a one-way ANOVA was used.

## 3. Results

A. Demographic data

The sample consisted of 259 H individuals and 292 G individuals. The sex distribution (male/female ratio) was more homogenous in the H (41%/59%) group than in the G (24%/76%) group. The most prevalent age group among H participants was between 41 and 50 years (30.5%), whereas, nearly 50% of the G participants were younger than 30 years (*p* < 0.001). Most H respondents had a high level of education (48%), whereas, 52% of G had finished only high school. The complete demographic data (including place of residence, marital status, and employment status) are presented in Table 1.

B. Risk factors for the development of chronic non-communicable diseases

The majority of survey respondents in the H group were nonsmokers (80%), never consumed alcohol (29%), sometimes consumed a diet rich in saturated fats and carbohydrates (65%), and had an average BMI of 24.3 kg/m^2^. They rarely control (measure) blood pressure (85%) or complete blood count (CBC)/serum biochemistry (67%), and had no habit of preventive doctor visits (46%). Among all of them, 50% had a positive family history of CVDs, and 5% had positive family history of PDs. Similarly, the majority of survey respondents in the G group were nonsmokers (66%), never consumed alcohol (42%), sometimes consumed a diet rich in saturated fats and carbohydrates (50%), and had an average BMI of 23.4 kg/m^2^. They rarely control (measure) blood pressure (89%) and had no habit of preventive doctor visits (41%), but their CBC/serum biochemistry was checked on every year (53%). Among all of them, 54% had a positive family history of CVDs, and 11% had positive family history of PDs (Table 2). The statistically significant differences between the groups included sex (women were more prevalent in the G group, *p* < 0.01), education level (lower education level in the G group, *p* < 0.01), and BMI (lower BMI in the G group, *p* < 0.05). The frequency of PA of different intensities (light, moderate, and high) among the H and G groups is shown in Table 3. Statistically significant differences between the groups were found for the moderate- and high-intensity categories (*p* < 0.05).

C. Comorbidities

**Figure 1:**
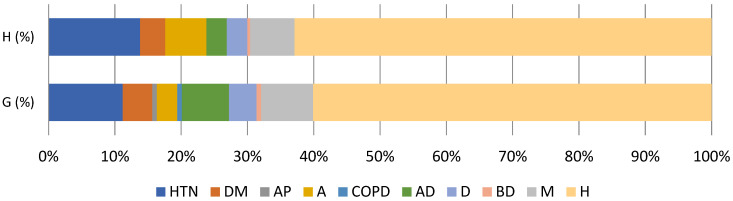
Prevalence of comorbidities in the H (total N = 259 (100%), top chart) and G (total N = 292 (100%), bottom chart) groups. HTN, hypertension; DM, diabetes mellitus type 2; AP, angina pectoris (stable); A, atherosclerosis; COPD, chronic obstructive pulmonary disease; AD, anxiety disorder; D, depression; BD, bipolar disorder; M, multiple comorbidities (HTN/DM + AD/D); H, healthy. There were 163 (62.9%) healthy individuals in the H group and 177 (60.7%) in the G group.

Of all respondents, 199/259 people from the H group (76.8%) and 218/292 people from the G group (74.7%) reported themselves as healthy (Table 2 and Figure 1). In the H group, the most prevalent condition was HTN (13.8%) followed by atherosclerosis (6.2%) and diabetes mellitus type 2 (DM, 3.8%). There were no reports of chronic obstructive pulmonary disease (COPD) and stable angina pectoris (AP). Regarding PDs, anxious disorder (AD) was equally reported as depression (D, 3.1%) followed by bipolar disorder (BD, 0.4%). Multiple comorbidities (cardiovascular and psychiatric, HTN/DM + AD/D) was observed in 6.7% respondents.

Similarly, in the G group, the most prevalent condition was HTN (11.3%) followed by DM (4.5%) and atherosclerosis (3.1%). COPD was equally reported as AP (0.7%). Regarding PDs, AD was reported by 7.2% respondents, which was followed by depression (4.2%) and BD (0.7%). Multiple comorbidities (cardiovascular and psychiatric, HTN/DM + AD/D) were observed in 7.9% respondents.

The respondents in the H group had significantly longer durations of disease, on average, by three years (*p* < 0.05). For pharmacotherapy and/or the occasional use of drugs, the majority of the respondents in both groups (~67%) used no drugs, thus, the risk of drug interactions was minimized (Table 2).

D. Characteristics of the hikers

Almost a quarter (24.7%) of our survey respondents from the H group had been hiking for between 5 and 10 years, 17% had been hiking for almost 20 years, and 12% of those in the H group had started hiking 2 years ago. Approximately 8% of our respondents had been hiking for more than 30 years (Table 2). Nearly 50% of the H group had been hiking a week prior. Regarding more serious hikes, 60.2% of them had had travelled high-altitude routes (above 2500 m) at least once in the last year. The majority (92%) did not experience any health problems and did not carry any drugs (67%) during hiking. When symptoms occur, the most common was excessive, nontolerant tachycardia (55% of the H individuals who reported health problems during hiking). They (66%) preferred going with a group of friends and usually keep in touch with people they meet on hiking trips (85%). When asked about their motivation for hiking, 75% of them stated all of its beneficial effects—mental health, PA, and staying outdoors, in nature. Regarding mental health, almost every respondent experienced improvements in several mental health spheres—a better mood (97%), a reduction in stress and anxiety (96%), and more energy at the beginning of the working week (88%) (Table 4).

E. Pharmacotherapy and side effects

**Figure 2:**
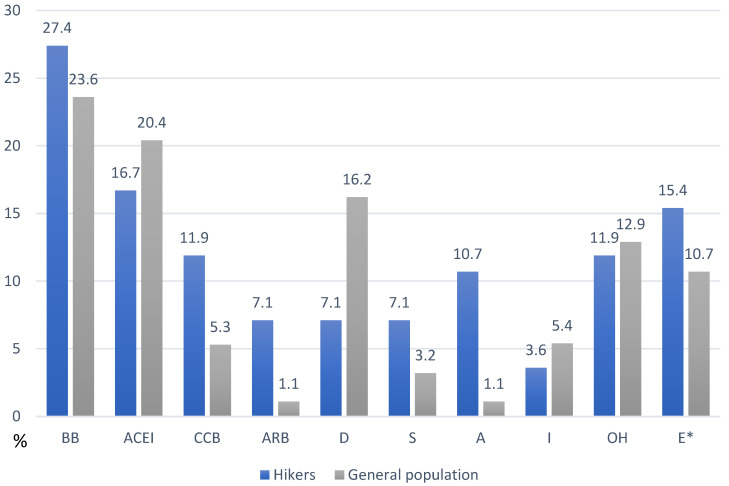
The frequency of cardiovascular drug consumption among hikers (left columns) and the general population (right columns). BB—beta blockers, ACEI—angiotensin-converting enzyme inhibitor, CCB—calcium channel blocker, ARB—angiotensin receptor blocker, D—diuretic, S—statin, A—acetylsalicylic acid, I—insulin, OH—oral hypoglycemic agent, E–Euthyrox^®^. *—the most prevalent drug class in addition to cardiovascular drugs and drugs used in diabetes mellitus. Data are presented in relation to the total number of people who used one or more drugs (93/292 [31.8%] in the G group and 84/259 [32.4%] in the H group).

**Figure 3:**
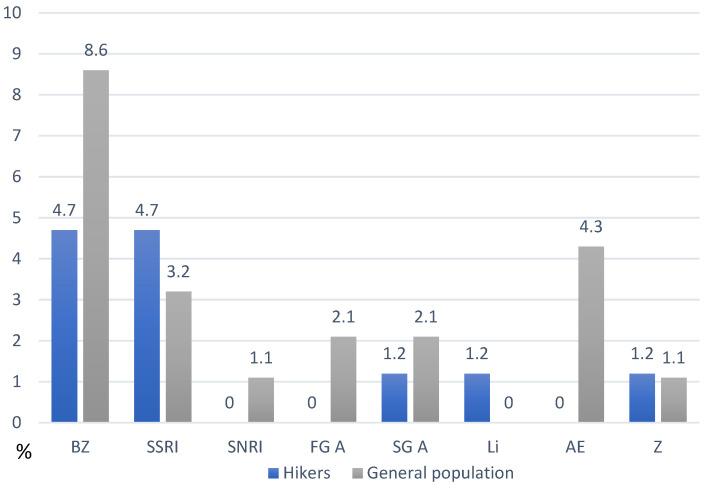
The frequency of psychotropic drug consumption among hikers (left columns) and the general population (right columns). BZ—benzodiazepines, SSRI—selective serotonin reuptake inhibitors, SNRI—serotonin and norepinephrine reuptake inhibitors, FG A—first generation antipsychotics, SG A—second generation antipsychotics, Li—lithium, AE—antiepileptic drugs, Z—hypnotics (‘Z’ drugs).

**Figure 4:**
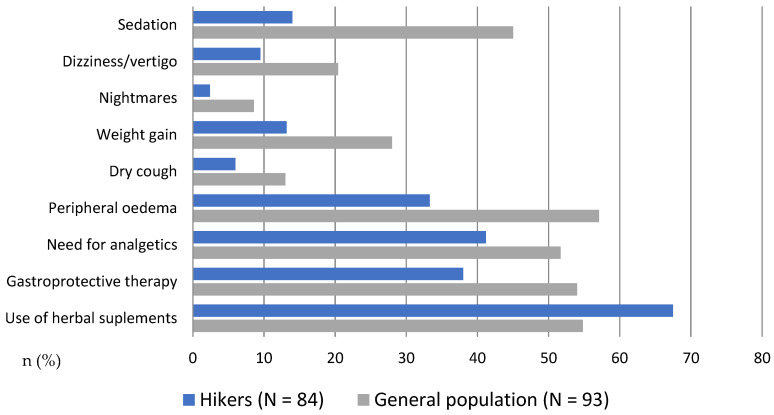
The frequency of reported drug side effects among hikers (blue columns) and general population (grey columns). The data are presented as relative numbers (percentage of the people who take chronic pharmacotherapy).

The drug classes and their incidences among respondents with CVDs and PDs are presented in Figure 2 and Figure 3. Among cardiovascular drugs, the most prevalent were beta blockers (27% in the H group, 24% in the G group; in both groups, >60% of those prescribed beta blockers were bisoprolol), ACEIs (17% in the H group, 20% in the G group; in both groups, >50% of those prescribed ACEIs were perindopril) and calcium channel blockers (12% in the H group, 5.5% in the G group; in both groups, >60% of those prescribed calcium channel blockers were amlodipine). The use of other cardiovascular drugs was less prevalent: diuretics (7% in the H group, 16% in the G group; in both groups, >30% of the prescribed diuretics were indapamide), statins (7% in the H group, 3% in the G group; in both groups, >50% of the prescribed statins were atorvastatin), and ARBs (7% in the H group, 1% in the G group, 50% in the prescribed ARB were losartan; in the G group, one respondent used telmisartan). The use of acetylsalicylic acid was significantly more prevalent in the H group (11% vs. 1%). The prevalence of drugs used for treating endocrine disease is also presented (Figure 2). Among the drugs used for PDs, the most prevalent were sedative-anxiolytic benzodiazepines (4.7% in the H group, 8.6% in the G group; in both groups, >30% of the prescribed benzodiazepines were bromazepam) and selective serotonin reuptake inhibitors (SSRIs) (4.7% in the H group, 3.2% in the G group; in both groups, >60% of the prescribed SSRIs were escitalopram). The only SNRI in one respondent in the G group was duloxetine. For mood stabilizers, four respondents (4.3%) in the G group used antiepileptics (valproate, carbamazepine), whereas, two participants in the H group (1.2%) used lithium. The prevalence of drugs used for psychotic disorders and the short-term treatment of sleeping problems is also presented (Figure 3). Among the common side effects of both groups (psychiatric and cardiovascular drugs), the most prevalent were peripheral edema (33% in the H group, 57% in the G group), sedation (14% in the H group, 45% in the G group), weight gain (13% in the H group, 28% in the G group), dry cough (6% in the H group, 13% in the G group), and dizziness/vertigo (9.5% in the H group, 20% in the G group). The occasional use of herbal supplements and gastro-protective, analgesic, and anxiolytic therapies is also presented in Figure 4.

## 4. Discussion

To our knowledge, this is the first pilot study in RS to cover the health overview of recreational hiking regarding comorbidities and associated pharmacotherapy, with an emphasis on the quantitative aspects of drug consumption and drug-related side effects between recreational hikers and the general population. This work also addresses which population has the greater disease burden and complications of common chronic diseases.

However, there was a difference in age between the groups, considering that nearly 50% of the respondents in the G group but only 9% of those in H group were younger individuals, defined as individuals between 18–30 years of age (Table 1). Younger people are generally healthier, have easier access to digital media, and spend more time on the internet, thus, our questionnaire, like other online surveys, could be easily assessed [22].

On the other hand, the community of hikers has grown continuously over the last two years. In 2021, the MAS consisted of 179 mountaineering clubs/societies throughout the RS, with 20,492 officially registered members [23]. The majority of them are, however, elderly (the most prevalent age group in range 41–50 years, accounting for 21% of the total number of MAS members). Since 56% of the MAS members are older than 30 years [23], there are continuous efforts by mountaineering associations to include children and young people in hiking. This approach is extremely important for the primary prevention of chronic non-communicable diseases, since patterns of risky behaviors and unhealthy habits may be adopted early in childhood [24]. Moreover, the fact that the majority of our older respondents had relatively little mountaineering experience is encouraging (Table 4), as it confirms that mountaineering is suitable for all ages, so more and more elderly choose this activity.

The complexities between individual hiker factors (comorbidities, age, and water/electrolyte intake) and environmental factors (extreme temperature and altitude) may contribute to the development of life-related, altitude-associated events, which have been documented in people with pre-existing conditions and in hikers [25]. For some hikers, the initial symptoms develop at higher altitudes, which may suggest hard-to-recognize medical conditions [26]. In our research, the majority of people did not experience any symptoms while hiking, except for excessive tachycardia, which can be considered an exaggerated physiological response to altitude or, in some cases, a lack of adequate physical condition. To prevent excessive symptoms of altitude sickness and other altitude-related symptoms, the official MAS regulation on the Health Protection of Mountaineers forces regular annual health check-ups for mountaineers, however, according to our experience, the response of hikers needs to be more common in practice. Prior to action, it is important to identify people with chronic pharmacotherapy, because the pharmacokinetics and pharmacodynamics of commonly used drugs can change at high altitudes [27], resulting in the higher occurrence of side effects. There was no difference in drug consumption between the groups. However, the frequency of risk factors (smoking, alcohol consumption, etc.) indicates that hikers practice healthier lifestyles and are more aware of the importance of PA for a healthy and long life.

The prevalence of at least one CVD was higher in the H group (23.5% in the H group and 19.5% in the G group). This is far more than other studies, which recorded the prevalence of at least one CVD among 7.4% [28] or even 5.8% [29] of ski mountaineers. The drastic discrepancy between these results, however, may be explained by the important age difference among participants in our study and studies from below. Specifically, those studies included participants from 8–77 years (mean age of 42.4 ± 12.7 years) [29], or even 6–76 years (mean age of 41 ± 14 years) [28], while we included only respondents of legal age (above 18 years). Moreover, we recorded a higher percentage of elderly (above 60 years)—15.8% compared to 12%, as observed in [29]. Here, we must state an important age difference between our H and G groups, which may lead to an improper interpretation and wrong conclusion of greater CVD burden in the H group. This is due to the unequal frequency of respondents above 40 years of age among the groups (68.7% in the H group and 38.6% in the G group). The prevalence of CVDs is greater with age, so this may be considered a sample error that we could not avoid.

Another reason for lower CVD in ski mountaineers [28] may be the higher percentage of people reported as physically active at least once a week (91.4%) compared to our H group, 32.8% for moderate PA). As for gender differences, males were dominant in both previous studies (above 70%), in contrast with ours (41.3%). The prevalence of CVDs was greater in males, but acute cardiovascular event, greater death rate, and worse prognosis were seen more frequently in females (58.7% in our study).

HTN was the most prevalent condition (14% and 11% for the H and G groups, respectively). According to a cohort study of 1279 hikers, the self-reported history of HTN was slightly lower—9.5% [30]. This greater uniformity with our results probably cannot be explained solely by a respondents’ age, anthropometric characteristics (participants above 40 years were 99.2% in [30] and 68.7% in our study), with BMI > 25 kg/m^2^ (77.4% in [22] and 38.6% in our study), or by the male predominance (92.5% in [30] and 41.3% in our study). Probably, this equality may be due to the presence of risk factors for HTN (alcohol consumption, unhealthy diet), which was prevalent in our study, but not discussed in detail in [30].

For most respondents, symptoms of the disease are controlled by pharmacotherapy (only 5% of the people in both groups received no antihypertensive therapy). The beta blocker bisoprolol was the most commonly used cardiovascular drug (25% of the people in both groups). Some time ago (1977–2003), beta blockers were recommended by the Joint National Committee on the Detection, Evaluation and Treatment of High Blood Pressure as a first-line treatment option for primary hypertension. However, the lack of evidence of reduced morbidity and mortality with respect to cardiovascular events made beta-blockers inferior to other classes of antihypertensive drugs [31]. The cardio-selective beta blocker bisoprolol is preferable for elderly with asthma, chronic obstructive pulmonary disease, or diabetes mellitus [32], a common comorbidity in both groups. Even though it can exacerbate the symptoms of peripheral claudication, it is not generally contraindicated in people with peripheral arterial disease (one respondent in the H group). The third generation (beta blockers with vasodilating properties, such as nebivolol/carvedilol) was also used by our survey respondents.

Among ACEI, perindopril was the most commonly used, both as monotherapy and in combination with thiazide diuretic indapamide. No patient on ACEI reported any renal comorbidities. CCBs were reported to be the third most common antihypertensive class, with amlodipine as the most prevalent. The tenfold greater prevalence of acetylsalicylic acid use in the G group can be explained by the broad spectrum of indications (in addition to CVDs) for which acetylsalicylic acid is used (as analgesics [in moderate pain, i.e., headache, abdominal pain during the menstrual cycle] or as an antithrombotic [i.e., secondary prevention of myocardial infarction]) [33].

Common side effects of cardiovascular drugs (peripheral edema due to CCB and dry cough/renal impairment due to ACEI) were present in both groups and were more common in the G group. This finding is in accordance with the known profiles of the side effects of those drugs [34]. Even though ACEI express better pharmacological profiles in people with metabolic diseases, thiazides may produce important side effects (drug-induced diabetes mellitus, hypercalcemia) when used in fixed combinations, but those effects have not been reported. There were no reports of peptic ulcers, GI bleeding, asthma attack, or nephrotoxicity induced by aspirin. None of the respondents reported myalgia, elevated liver enzymes, or rhabdomyolysis, which can be seen as side effects of statins (rosuvastatin) [35].

PDs were more prevalent in the G group (12%) than in the H group (6%), which can be explained by the positive influence of outdoor activities on individuals’ mental health and cognitive functions [36]. In a recent Polish study [37], young people among adventure hikers reported high levels of life satisfaction, meaning of life, and positive emotions, reporting in various wellbeing scales. Moreover, hikers performing long distances (100–5500 km/year) have excessive intrinsic will to overcome new challenges, find physical boundaries, and experience the state outside their comfort zone [38], which is crucial for the overall wellbeing. The latest systematic research involving more than 8500 records confirmed the positive relationship between long-distance walking and mental health, suggesting that long-distance walking may be a form of psychotherapy [39]. In both groups that we reported, anxiety was the leading cause, thus, the most commonly used drugs were benzodiazepines (anxiolytics). However, the recreational consumption of benzodiazepines was rather prevalent, which is worrying because of their addictive nature, high rate of sedation, and interaction with other drugs. In the latest article [40], benzodiazepines are the most commonly inappropriately prescribed medications (according to the Beers criteria list of medication guidelines that help healthcare professionals prescribe safe medications for adults over the age of 65) in elderly with CVDs, which may contribute to increased mortality in that population [41]. In our study, notably less anxiolytic consumption was reported in the H group. This is very important, considering that RS is known for excessive anxiolytic consumption (in 2022, 5.2 million boxes of anxiolytics were sold in the RS) [42]. Therefore, recreational hiking can be one of the easiest ways to decrease the socioeconomic burden of those drugs. On the contrary, the use of antidepressants (SSRIs) was higher in the H group. This could be expected, since SSRIs are used as a drug of choice (DOC) in the initial phase of treatment. They are used as first-line antidepressant due to their great therapeutic efficacy, additional anxiolytic properties after long-term use, and the lack of debilitating, mostly anticholinergic side effects (like sedation or constipation). In most cases, after 2–4 weeks of use, these drugs cause improvement in about 45% of depressive patients, but it is recommended to continue this therapy for at least 6–12 months [42]. The fact that the H group used more SSRIs than the G group probably means that they are in a state of stable disease with no recent change in medication, or they may have a mild form of the disease that can be treated successfully with SSRIs. Moreover, the anxiolytic properties of SSRIs reduce the risk for hyperventilation that might be a predisposition towards acute mountain sickness [42].

The negligible number of hikers reported lithium as a DOC for manic episodes of BD, and there were no reports in the G group (despite higher prevalence of BD compared to the H group). This may be due to the ‘stigmatizing’ effect of lithium treatment, as it is perceived as a medication for severely ill patients, so our respondents (mostly younger) were embarrassed to report [43].

On the other hand, the low prevalence of antipsychotic consumption is expected and medically justified because of their side effects (ECG changes: the prolongation of the QT interval). This is described as a possible cause of sudden cardiac death syndrome in the mountains [44,45]. For those people, an adequate pre-hike assessment of cardiovascular risk factors must be made [12].

In the modern age, digital technologies and sedentary lifestyle represent unquestionable challenges to mental health, especially in the era after the SARS-CoV-2 pandemic [6]. Today, maintaining mental health is a global problem, which people of all ages are constantly facing. According to UNICEF research [46], younger and adolescents are particularly at risk, as they may have various social issues, such as social exclusion, problems with peer group assimilation, and inadequate generation and/or direction of their cognitive potential. However, the etiology of mental problems in the elderly often derives from the use of chronic pharmacotherapy, which, in addition to the side effects on the somatic system, can also reduce psychomotor activity, emotions, and other effects. Regardless of age, the degree of wellbeing and prosperity of a country is directly correlated with the preservation of the mental health of the individuals, so the public activities should be directed towards prevention of mental disorders. Moderate PA like hiking, especially if it is carried out in the nature, is recognized by professionals as an effective way to improve quality of life [46].

Today, various protocols include PA in managing different diseases, both from a preventive and therapeutics point of view [47]. The vast majority of respondents, especially those with frequent PA, report no chronic diseases or everyday drug use, which suggests the influence of non-pharmacological measures in the prevention of chronic diseases, such as diabetes mellitus, obesity, hypertension, and clinical course and the outcome of PDs. In addition to wellbeing, studies indicate that hikers are generally better prepared for the health-related problems they encounter in nature [48]. Important aspects of outdoor activities (such as hiking) include the distraction of children and young people from digital media, closed spaces, and often extremely poor air quality, as observed in various cities of the RS, most notably in the capital, Belgrade. Also, the global prevalence of obesity and metabolic syndrome among children and young people is increasing alarmingly [49], which is seen in the RS too [50]. Unfortunately, this may be confirmed by our study, as the majority of respondents from the G group (mostly younger, 18–30 years) reported no (58.9%) or less than two days a week (21.9%) of moderate PA. They also reported no (74.7%) or less than two days a week (14%) of high PA. This apparent discrepancy is expected, since hiking is considered as moderate-to-high PA, and there were no reports of engaging in other types of sports or regular recreational activities from the respondents in the G group.

Ball sports are still the most popular PA among school children. The engagement in one of the ‘’big 4′’ (football, basketball, volleyball, and handball) during and after school classes is important for their physical and psychosocial development but is also responsible for a big share of reported sport-related injuries, for example contusions of the ankle, fingers, and upper extremities [51,52]. Regarding trauma, hiking too is not fully safe, since there are some reports of injuries, frostbite, and hypothermia, but those are very rare (7%) and depend on several factors, such as backpack volume, BMI > 30 kg/m^2^, and previous reports of injuries [53,54,55], so it may be considered safer than other sports. From a public health point of view, the advantages of hiking go far beyond individuals. This means that the improved psychophysical health of an individual, through social interactions and long-term, long-distance walking, may highlight the importance of healthy lifestyle habits and, after all, create a healthier environment for this and future society [56].

This pilot study has several limitations. The major limitations of this research include self-reports of the disease (with no medical documentation or medical history attached), the small sample size (1.26% of the official total number of MAS community in 2021), younger population in the G group, and the observational methodology. However, this work represents pioneering action for the engagement of different people, even with co-morbidities, into hiking activities.

## 5. Conclusions

In conclusion, despite the age gap between the groups, a similar incidence of CVDs was observed in hikers and in the general population, whereas, PDs were more prevalent in the general population, so our hypothesis was partly confirmed. Drug consumption and the side effects of chronic pharmacotherapy also share similar patterns. To clarify the influence of hiking on health in the RS, a larger sample size, a follow-up period for the evaluation of hiking effects, and stronger statistical tools are necessary. However, general points from this pilot study are important, since they highlight hiking as an outdoor activity that is highly important for the prevention of psychosomatic diseases and should be considered as official recommendations by the general practitioners, especially for patients at risk.

That being said, the results of this research could introduce new recommendations in the official guidelines for the nonpharmacological management of CVDs regarding the extent and the type of recommended PA. Additionally, the results obtained may aid in the implementation of hiking as a recommended PA by primary healthcare providers from the perspective of preventing PDs, i.e., reducing the need for psycho-pharmacotherapy, which is suitable for people of all ages.

## Figures and Tables

**Table 1:** Sociodemographic characteristics of the study population.

Demographic Characteristics	Hikers	General Population
	N (%)	
Age	s	
18–30	23 (8.9)	145 (49.7)
31–40	58 (22.4)	34 (11.6)
41–50	79 (30.5)	64 (21.9)
51–60	58 (22.4)	37 (12.7)
61–70	33 (12.7)	12 (4.1)
>70	8 (3.1)	0
Sex (Male/Female)	107 (41.3)/152 (58.7)	69 (23.6)/223 (76.4) *
Education		
Primary school	1 (0.4)	6 (2.1)
High school	61 (23.5)	152 (52.1)
Higher school	29 (11.2)	11 (3.8)
Faculty	125 (48.3)	103 (35.3)
PhD studies	43 (16.6)	20 (6.8)
Employment (Yes/No)	213 (82.2)/46 (17.8)	155 (53.1)/137 (46.9)
Marriage status		
Unmarried	83 (32)	156 (53.4)
Married	103 (39.8)	92 (31.5)
Divorced	39 (15.1)	23 (7.9)
Widow	11 (4.2)	6 (2.1)
Extramarital union	23 (8.9)	15 (5.1)
Living place		
City	212 (81.9)	241 (82.5)
Suburb	29 (11.2)	34 (11.6)
Countryside	18 (6.9)	17 (5.8)

*—statistical significance.

**Table 2:** Presence of risk factors for the development of chronic non-communicable diseases among the study population. 0—complete blood count.

Risk Factors	Hikers	General Population
	N (%)	
Smoking status		
Nonsmoker	208 (80.3)	193 (66.1)
<1 year	3 (1.2)	10 (3.4)
Several year	10 (3.9)	24 (8.2)
More than a decade	38 (14.7)	65 (22.3)
Alcohol consumption		
Never	75 (29)	122 (41.8)
Once/week	71 (27.4)	76 (26)
Several times/week	55 (21.2)	30 (10.3)
Once/month	58 (22.4)	64 (21.9)
Unhealthy diet		
No	40 (15.4)	27 (9.2)
Sometimes	168 (64.9)	147 (50.3)
Often	29 (11.2)	95 (32.6)
Every day	9 (3.5)	23 (7.9)
BMI		
Underweight (<18.5)	7 (2.7)	15 (5.2)
Normal (18.5–24.9)	152 (58.7)	189 (64.7)
Overweight (25.0–29.9)	81 (31.3)	64 (21.9)
Obese (>30.0)	19 (7.3)	24 (8.2)
Blood pressure measurements		
Rare	221 (85.3)	259 (88.7)
Once/week	28 (10.8)	22 (7.5)
Several times/week	10 (3.9)	11 (3.8)
CBC/serum measurements		
Rare	173 (66.8)	136 (46.6)
Once/year	83 (32)	156 (53.4)
Several times/year	3 (1.2)	0
Doctor visiting		
None	96 (46)	83 (40.7)
Once/year	73 (36.1)	72 (35.3)
Several times/year	36 (17.9)	49 (24)
Comorbidities		
Cardiovascular diseases	61 (23.5)	57 (19.5)
Psychiatric diseases	17 (6.6)	35 (12)
Average duration of the disease (years) ($\bar{x}$ ± SD)	11.9 ± 10.5 *	8.4 ± 8.7 *
Drug interactions		
No therapy	175 (67.6)	199 (68.2)
One drug	35 (13.5)	45 (15.4)
Two drugs	34 (13.1)	30 (10.3)
More than three drugs	15 (5.8)	18 (6.2)
Family history		
Cardiovascular diseases	134 (51.7)	157 (53.8)
Psychiatric diseases	14 (5.4)	33 (11.3)

*—statistically significant.

**Table 3:** Frequency of physical activity (light, moderate, or high) among our groups (first number, hikers; second number, general population).

Physical Activities Intensity/Frequency	Light (%)	Moderate (%)	High (%)
Everyday	71.4/65.1	12.7/5.5	1.5/0.7
5–6 days	11.2/12.7	4.6/2.4	1.3/1
3–4 days	12/11.6	25.5/11.3	12.7/9.6
<2 days	4.6/9.2	32.8/21.9 *	29.7/14 *
Never	0.8/1.4	24.7/58.9 **	54.8/74.7 **

* *p* < 0.05, ** *p* < 0.01.

**Table 4:** Relevant characteristics of the hiker’s action (from hiker experience).

Relevant Characteristics of the Hikers (N = 259 (100%))	N (%)
Average years of hiking experience. *n*—year (s)	
*n* ≤ 2	30 (11.6)
2 < *n* ≤ 5	75 (29)
5 < *n* ≤ 10	64 (24.7)
10 < *n* ≤ 20	45 (17.4)
20 < *n* ≤ 30	23 (8.9)
30 < *n* ≤ 40	11 (4.2)
40 < *n* ≤ 50	8 (3.2)
50 < *n* ≤ 60	3 (1.2)
Time of last hiking action	
Last week	123 (47.5)
Last month	57 (22)
Several months ago	66 (25.5)
More than a year	13 (5)
Main reason/motivation for hiking	
Mental health	7 (2.7)
Physical activity	12 (4.6)
Stay in nature	46 (17.8)
All of the above	194 (74.9)
Participation in high mountaineering (>2500 m) actions during the last year	
Once	65 (25.1)
2–5	65 (25.1)
More than five times	26 (10)
I do not participate in actions > 2500 m altitudes	103 (39.8)
Health problems during the action	20 (7.7)
Symptoms	
Excessive/disturbing tachycardia	11 (55)
Shortness of breath	7 (35)
Excessive sweating	3 (15)
Nausea/vomiting/GIT disturbances	2 (10)
Anxiety	1 (5)
Carrying the drugs on the action	85 (32.8)
Hiking company (alone/group of friends)	89 (34.4)/170 (65.6)
Mood after the hiking (better/same)	252 (97.3)/7 (2.7)
Reduction in stress and anxiety after hiking	249 (96.1)
Keeping in touch with people met on hiking trips	219 (84.6)
More energy at the beginning of the working week	229 (88.4)

## Data Availability

The data that support the findings of this study are available on request from the corresponding author. The data are not publicly available due to privacy or ethical restrictions.
